# Postural Instability Detection: Aging and the Complexity of Spatial-Temporal Distributional Patterns for Virtually Contacting the Stability Boundary in Human Stance

**DOI:** 10.1371/journal.pone.0108905

**Published:** 2014-10-08

**Authors:** Melissa C. Kilby, Semyon M. Slobounov, Karl M. Newell

**Affiliations:** 1 Department of Kinesiology, The Pennsylvania State University, University Park, Pennsylvania, United States of America; 2 Center for Sport Concussion Research and Services, The Pennsylvania State University, University Park, Pennsylvania, United States of America; University of California, Merced, United States of America

## Abstract

Falls among the older population can severely restrict their functional mobility and even cause death. Therefore, it is crucial to understand the mechanisms and conditions that cause falls, for which it is important to develop a predictive model of falls. One critical quantity for postural instability detection and prediction is the instantaneous stability of quiet upright stance based on motion data. However, well-established measures in the field of motor control that quantify overall postural stability using center-of-pressure (COP) or center-of-mass (COM) fluctuations are inadequate predictors of instantaneous stability. For this reason, 2D COP/COM virtual-time-to-contact (VTC) is investigated to detect the postural stability deficits of healthy older people compared to young adults. VTC predicts the temporal safety margin to the functional stability boundary ( =  limits of the region of feasible COP or COM displacement) and, therefore, provides an index of the risk of losing postural stability. The spatial directions with increased instability were also determined using quantities of VTC that have not previously been considered. Further, Lempel-Ziv-Complexity (LZC), a measure suitable for on-line monitoring of stability/instability, was applied to explore the temporal structure or complexity of VTC and the predictability of future postural instability based on previous behavior. These features were examined as a function of age, vision and different load weighting on the legs. The primary findings showed that for old adults the stability boundary was contracted and VTC reduced. Furthermore, the complexity decreased with aging and the direction with highest postural instability also changed in aging compared to the young adults. The findings reveal the sensitivity of the time dependent properties of 2D VTC to the detection of postural instability in aging, availability of visual information and postural stance and potential applicability as a predictive model of postural instability during upright stance.

## Introduction

Falls, in particular among the elderly, are a serious threat to their functional mobility in activities of daily living [1]–[2]. It is crucial to understand what mechanisms and processes cause falls in the elderly. To do so, there is a pressing need for fall prediction and detection algorithms [3]–[5] and more generally for methods that identify groups of people at a higher risk of falling due to disease or aging [1], [6]–[8]. Previous research has considered numerous factors such as diabetes mellitus, history of falls, fear of falling, visual impairment, depression or postural control deficits (increased postural sway) as fall predictors in the elderly [9]–[12]. Postural sway, that is, the amount of whole body postural motion (center-of-pressure (COP) or center-of-mass (COM) fluctuations) in quiet standing is a well-established measure in the field of motor control [13]–[15]. It has been shown that healthy aging progressively increases postural motion when standing still over time periods of 20-30 s up to several min [2], . However, the commonly reported overall postural motion appears to be an inadequate predictor of instantaneous stability in quiet upright stance, because even if an overall greater amount of postural motion happens to coincide with reduced overall stability, this measure may not provide an estimate of the level of instantaneous stability [17]–[20]. Yet, quantification of the instantaneous postural stability is pivotal in developing applicable on-line falls prediction algorithms.

Mechanical inverted pendulum models of upright stance [18], [20] and virtual time-to-contact (VTC) approaches [21]–[24] appear to be more relevant in the regard of dynamic prediction. VTC quantifies the temporal proximity to the stability boundary, which is commonly defined geometrically as the outside edge of the feet that coincides with the limits of the base of support [18], [20]. Thus, VTC is highly relevant to postural stability from a mechanical point of view. The general emphasis in this view is the temporal safety margin to the stability boundary based on the current spatial position, velocity and acceleration of the COM or COP [22]–[23], [25] rather than the spatial and/or temporal departure from a presumed fixed point in the center of the postural stability region [15]. A direct implication is that a close position of the COM to the stability boundary together with a high velocity away from the nearest boundary can indeed reflect a relatively stable state. Among older people VTC has been shown to be a viable predictor of the instant of taking a step after a perturbation [25] and Slobounov et al. [22] also showed that 2D VTC was reduced with aging due to increased postural motion that takes place within a reduced stability boundary region [26].

The novelty of our study here was to implement the 2D VTC approach of Slobounov et al. [22] as a predictive model of postural instability in the elderly using dynamic quantities of VTC that have not been previously considered. More specifically, we aimed to quantify not only the temporal proximity to the stability boundary, but also to address the related fundamental question as to the direction of postural motion in which instability is increased [5], [27]–[28]. The directional information is critical in characterizing weaknesses or limitations of the postural control system and has the potential to being a direct indicator of an increased risk of falls into specific directions [3], [5]. To extract the directional information, we analyzed the spatial location on the 2D boundary of the stability region at which the virtual second order trajectory of COP or COM intersected the boundary. The stability boundary was divided into different segments in order to perform a distributional analysis of the probability of virtual contacts and the associated magnitude of VTC across the boundary segments. These polar distributions hold theoretical and clinical implications because actual and simulated falls have been reported with the indication of the direction of falling [3]–[5].

To our knowledge previous studies of quiet upright stance have solely analyzed postural responses more generally in anterior-posterior (AP) and medial-lateral (ML) directions, showing that AP usually exceeds ML motion [15], [29]–[30]. However, a more direct examination of postural instability in specific directions, for example, in the forward, backward or forward sideways directions has mostly been carried out in perturbation or leaning studies [23], [27]–[28]. Our study examined this new feature of VTC in young and old adults through removing visual information feedback [6] and enhancing postural motion near the lateral stability boundary by increasing the loading of one leg [31]–[33]. Furthermore, a dynamic sway condition allowed us to test the effect of this in contrast to the typical quiet stance condition. Of particular interest was whether the dynamic condition would channel the minimum VTC to a lower level than occurs in quiet stance through being dynamical closer (in a temporal limit sense) to the stability boundary.

The analysis of the time-dependent structure of VTC was included into the here introduced predictive model of postural instability as it has long been recognized that the temporal structure of postural adjustments quantifies a critical, non-mechanical property of postural stability [19], [34]–[35]. A reduced temporal structure, commonly termed complexity, is traditionally interpreted as a functional decline of the regulation of the postural control system – a feature that would enhance the likelihood of losing postural stability. Previous research has shown that the age related increase of postural motion in quiet stance occurs concomitantly with a reduction in the time- and frequency-dependent structure of the postural motion [35]–[37]. These patterns of change in the motor control of posture with aging have been shown to relate to the *loss of complexity* of the output of the motor system [36], [38] in that there is an age-related inverse relation between the dispersion and structure/complexity of postural motion [34]–[35], [37], that is hypothesized to be dependent on the task and the emergent attractor dynamics [36].

Here we investigate whether aging reduces the complexity of the spatial-temporal distributional patterns for virtually contacting the postural stability boundary. To study the complexity of VTC we applied the Lempel-Ziv-Complexity (LZC) algorithm [39]. As opposed to the generally applied non-linear time series analysis tools in the field of postural control [19], [34]–[35], [37] we applied LZC as this algorithm is applicable for on-line monitoring [40], requires a shorter minimum data length and finally the computational cost is lower. The basic idea of this algorithm is to detect repetitive patterns in a given sequence and dates back to finding algorithms to compress any given data set and, therefore, saving storage space. It is interpreted that if there are such recurrent patterns, there is redundant information and the shortest description of the critical information contained in the sequence must be shorter than the sequence length and thus can be compressed. A lower information content/complexity or higher consistency/structure links to increased predictability. Recognizing the degree of predictability of a system is an ambitious goal in many research fields [41], in that: 1) quantifying the predictability may correct the assumption of an at first sight random looking human process [6], [42] and 2: improving the ability to predict in our case human postural performance through algorithms may help to reveal in advance extended periods of postural instability based on previous performance.

The primary purpose of this study was to validate and extend 2D VTC as an approach to construct an applicable predictive model of postural instability during upright stance in the elderly. Therefore, aside from investigating the effects of aging, we also tested the effects of the availability of visual information (vision and no vision) and different loadings of the legs on the spatial-temporal distribution and complexity properties of VTC during two footed upright side by side stance. We hypothesized that VTC depends on age, and that this dependency would be influenced by the visual feedback condition and weight distributions between the legs. We predicted that: 1) the removal of visual information and aging would decrease the area of the stability region and the magnitude of VTC; 2) with aging, the withdrawal of vision and the manipulation of the weight loading on each foot would produce less stable spatial-temporal distributional patterns for virtually contacting the stability boundary; and 3) that the complexity of VTC dynamics will be reduced by aging, the withdrawal of visual information and the unequal load weighting on the legs.

## Methods

### Participants

Two age groups were recruited for this study, one group of twelve young adults (22.2±2.6 years, 5 males and 7 females) and one group of twelve old adults (69.7±2.3 years, 6 males and 6 females). All participants were self-reported non-fallers. In addition, based on self-report all participants were free from any neurological or neuromotor disorders and musculoskeletal injuries that could affect balance. The experimental protocol was approved by the Institutional Review Board of the Pennsylvania State University. After giving written informed consent, participants started with the experimental procedures.

### Apparatus

Whole body motion capture was realized using Qualisys Track Manager Software (Qualisys AB, Gothenburg, Sweden) and six ProReflex cameras that tracked the 3D coordinates of 22 passive reflective skin markers. Ground reaction force and moment signals were collected using two adjacent AMTI (American Mechanical Technology, Inc., Watertown, MA) strain gauge force platforms. The kinematic and kinetic data were synchronized and sampled at 100 Hz.

### Experimental procedures

There were two manipulations: vision and leg weight loading. Each loading condition was performed with and without vision (eyes open and eyes closed). The 4 different load weightings of the legs were: approximately equal weight on both feet (EqWe), more weight on the left leg (LWe) or on the right leg (RWe), and dynamically shifting the weight between legs during the trial (Dyn). Except for the dynamic trials, the task goal was to stand as still as possible for the entire duration of the trial. For the dynamic condition a 0.15 Hz metronome was implemented as an acoustic cue for shifting load on the feet. When shifting the weight to one leg, participants were asked to load one leg as much so that the posture was most comfortable. The trunk, however, should not be bent sideways.

Prior to data collection markers were attached to the skin at the following landmarks: Distal Phalanges, 5^th^ metatarsal, heel, lateral malleolus, lateral femoral epicondyle, greater trochanter, iliac crest, acromion process, lateral humeral epicondyle, dorsal wrist (between radial and ulnar styloid), and the lateral aspect of the head (anterior to ear canal). Participants were then asked to adopt a comfortable double leg standing posture on both force platforms (one foot on each of the two platforms) with the feet approximately hip width apart. A trace of this foot position was taken, so that the foot placement was the same for each trial.

Initially, the functional stability boundaries with eyes open and eyes closed were recorded. Participants were asked to maximally lean forward, backward and to either side without raising heels [22]. Subsequently the experimental trial blocks (3 trials of 30 s each condition) were conducted.

### Data processing

Data were processed in Matlab (MathWorks, Natick, MA). Postural center of pressure (COP) excursions were derived from the digitally low-pass filtered (10 Hz cutoff) ground reaction force and moment time series and whole body center of mass (COM) excursions were derived from the low-pass filtered (6 Hz cutoff) marker coordinates. The COM position was calculated as the weighted sum of segmental center of mass positions. We modeled the human body as 13 rigid segments (head, the upper arms and forearms/hands, thorax/abdomen, pelvis, and thighs, shanks and feet) using constant Dempster's body segment parameters to be consistent with previous work [15].

### Data analysis

**Figure 1:**
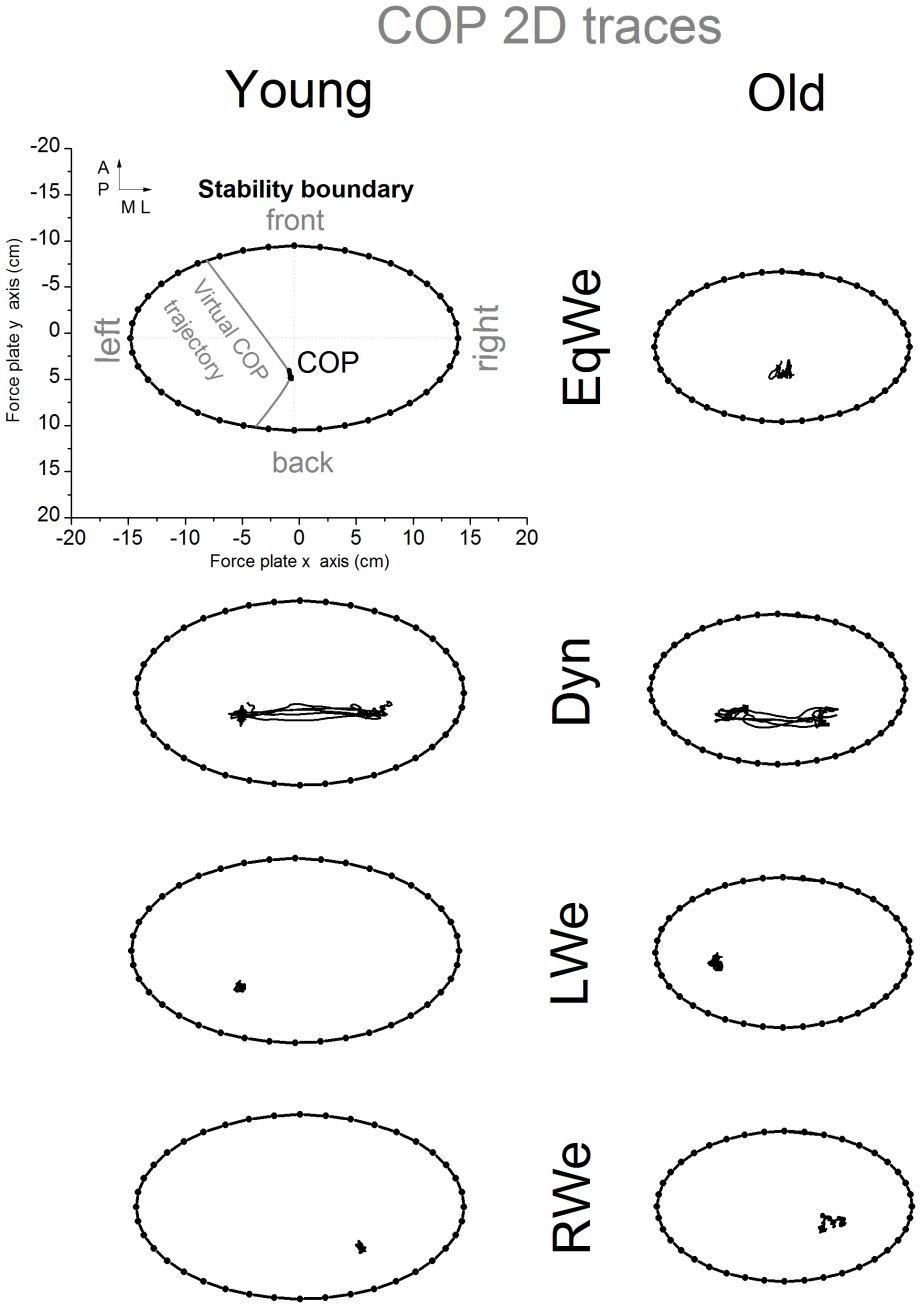
In the upper left panel the 2D COP path of one single representative trial with normal vision, the respective polygon representation of the 2D functional stability boundary and two virtual COP trajectories at arbitrary time instants are illustrated in original aspect ratio, resolved in the force platform coordinate system. Each boundary segment represented one specific direction in relation to the COP (e.g. front or back segments). Additional representative 2D traces of the COP with the functional stability boundary as a function of age and loading (with normal vision) are also illustrated.

The magnitude of the COP stability boundary was assessed through the area of the stability boundary model (Figure 1) and the amount of COP motion through the 2D path length. The analysis of the COM motion was limited to the motion in anterior-posterior (AP) and medial-lateral (ML) directions. Input data for VTC calculation in custom written Matlab code were the 2D position of the COP or COM along with the instantaneous velocity and acceleration vectors, respectively. In addition, the matching experimental boundary trial (for example boundary trial with removed vision for all no vision trials) was loaded simultaneously.

From this boundary trial a multi-segment polygon consisting of 40 line segments (Figure 1) was derived to model the coordinates of the functional stability boundary. The extrema of the functional stability boundary ellipse were based on the maximum motion of the COP or COM to the front, back and either side during the experimental boundary trials. Each segment of the boundary spans a sector of 9°. The supplementary video animation (Video S1) can be accessed for visualization of how the functional stability boundary was modelled.

Subsequently, the virtual time (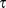) the COP or COM would need to contact with the stability boundary if it were to continue from the current position (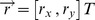) with instantaneous initial velocity (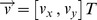) and instantaneous constant acceleration (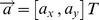) was extracted. Thus, we used a second order approximation to the virtual trajectory as it has been proposed to more appropriately represent the postural dynamics [17], [22].

The VTC (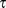) at each time instant (30 s at 100 Hz) was computed as follows [Haibach et al. 2007]. Let (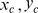) denote the point on the stability boundary where the virtual trajectory intersects it for the first time. If the end points of the corresponding boundary line segment are (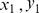) and (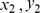) the slope (s) of the line connecting the two points is 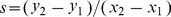
(1)Assuming constant slope in the differential segment between (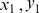) and (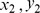), the slope can also be computed as 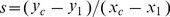
(2)


Assuming a point mass model for the COM and constant acceleration, the point of virtual contact can be written as, 
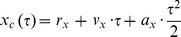
(3)




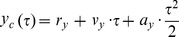
(4)


Substituting 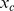 and 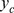 from equations 3–4 in 2, and equating it to 1, gives a quadratic equation in 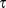. VTC (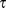) is the lowest positive solution of this quadratic equation. In the case where both velocity and acceleration were zero, VTC would be infinity.

The mean and minimum values of VTC were computed. Furthermore, the VTC time series was decomposed into as many time series as boundary segments, yielding a separate VTC time series for each of the 40 boundary segments. This representation related the VTC properties to the spatial location for virtually contacting the stability boundary. On this basis, we studied the distributional patterns across boundary segments for the mean VTC magnitude and the probability of virtual contacts.

The sequence of the virtually contacted boundary segments (Boundary) over time was further processed with non-parametric tools. We computed the algorithmic information theory-based Lempel Ziv complexity (LZC) of these sequences [39]. This complexity measure characterizes the time evolutionary development of spatial-temporal patterns in nonlinear systems. To our knowledge this measure has previously not been used for analysis of postural sway data. However, it has been extensively used for analyzing other physiological measures, such as EEG data [40]. LZC is based on the number of distinct subsequences contained in the given sequence when scanning the finite sequence from left to right. The reader can refer to Zhang et al. [40] for a detailed description of the implemented LZC code. According to Kaspar and Schuster [43] the LZC was normalized to the upper theoretical limit that is based on the sample length and number of symbols contained in the sequence. Higher LZC values are indicative of greater complexity and the normalized LZC in biological signals is usually less than 1 [40].

### Statistics

The statistical analysis was performed using an Age (2 levels) by Vision (2 levels) by Loading (4 levels) ANOVA with repeated measures on the last two factors. The significance level was set at p<0.05. Post hoc pairwise multiple comparisons were made with the Bonferroni correction procedure. We used R software for the statistical analysis.

## Results

### COP displacement and COP stability boundary

**Figure 2:**
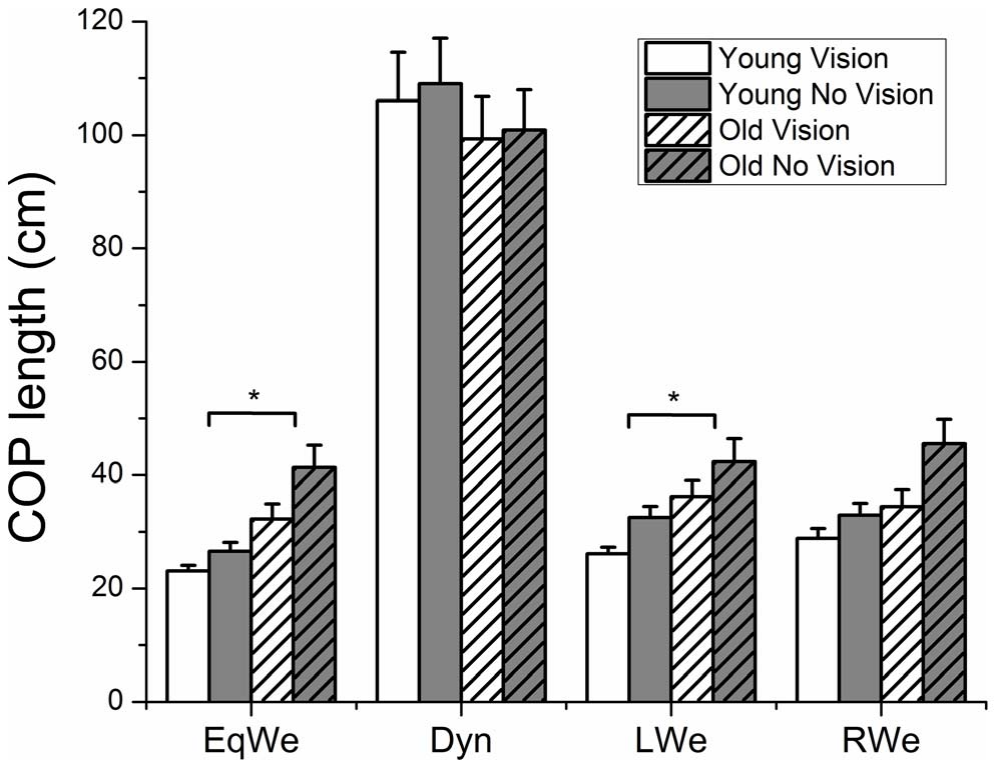
Group mean ± SE (N = 12) 2D COP path length as a function of age, vision and loading. The asterisks * illustrate the significant age × loading interaction with regard to age.

Figure 2 shows the mean COP path length as a function of age, vision and leg loading condition. There were main effects of vision (F_1,22_ = 20.98, p<0.01), loading (F_1.09,24.15_ = 234.72, p<0.01), and a significant age × loading interaction (F_1.09,24.15_ = 3.80, p<0.05).

Post hoc analysis showed that COP path length significantly increased under no vision. The age × loading interaction showed that COP path length only significantly increased for the old compared to the young group during the EqWe and LWe conditions. Further the age × loading interaction revealed that for both age groups COP path length was highest under Dyn compared to all other conditions. For the young adults the COP path length was also higher under either RWe or LWe in contrast to EqWe.

The functional COP stability boundary was significantly reduced with aging (F_1,22_ = 33.63, p<0.01) and when vision was removed (F_1,22_ = 36.10, p<0.01). Figure 1 shows representative 2D COP displacement traces for each of the 4 leg loading conditions with the respective stability boundary.

### Standard statistical properties of VTC (using COP)

**Figure 3:**
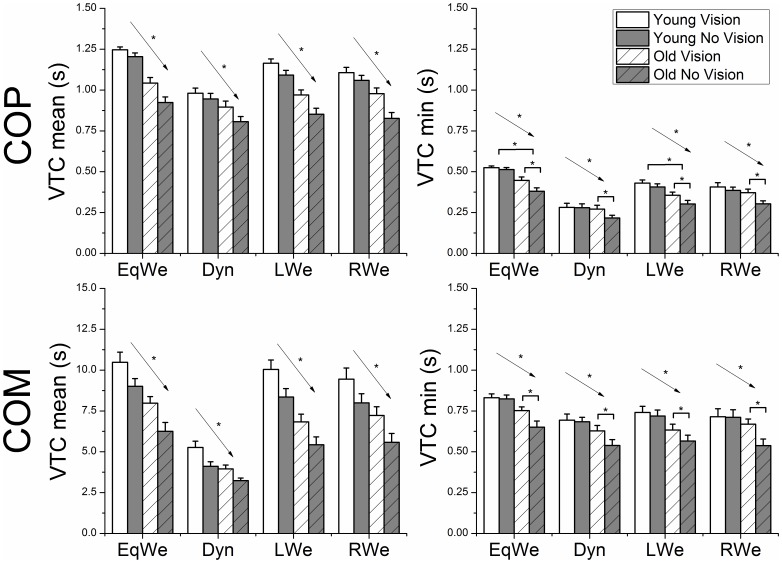
Group mean ± SE (N = 12) VTC mean and minimum values for both COP and COM as a function of age, vision and loading. The asterisks * illustrate the significant age × vision interaction with regard to vision and the age × loading interaction with regard to age. The arrow with asterisk indicates significant main effects of age and vision.

The upper two panels of Figure 3 show the mean VTC mean and min values using the 2D COP data. The main effects of age (VTC min F_1,22_ = 11.97, p<0.01; VTC mean F_1,22_ = 23.92, p<0.01), vision (VTC min F_1,22_ = 22.60, p<0.01; VTC mean F_1,22_ = 60.92, p<0.01), loading (VTC min F_3,66_ = 99.54, p<0.01; VTC mean F_3,66_ = 82.82, p<0.01), and the age × vision (VTC min F_1,22_ = 8.53, p<0.01) and age × loading (VTC min F_3,66_ = 3.40, p<0.05) interactions were significant.

VTC mean values generally decreased with the withdrawal of vision, whereas VTC min decreased with the withdrawal of vision only for the old adults. The VTC mean values were significantly reduced for the old compared to the young group. In addition, the VTC min age × loading interaction revealed that the VTC min values were only significantly reduced for the old compared to the young adults during EqWe and LWe. Further, post hoc analysis of the main effect of loading for both VTC mean and min values showed that either LWe or RWe reduced the VTC values compared to EqWe. Dyn had the lowest VTC values compared to the remaining conditions.

### Standard statistical properties of VTC (using COM)

The lower two panels of Figure 3 show the mean VTC mean and min values using the 2D COM data. The main effects of age (VTC min F_1,22_ = 8.68, p<0.01; VTC mean F_1,22_ = 16.12 p<0.01), vision (VTC min F_1,22_ = 12.78, p<0.01; VTC mean F_1,22_ = 65.33 p<0.01), loading (VTC min F_3,66_ = 21.23, p<0.01; VTC mean F_2.08,45.94_ = 121.65 p<0.01), and the age × vision (VTC min F_1,22_ = 8.26, p<0.01) and age × loading (VTC mean F_2.08,45.94_ = 121.65 p<0.01) interactions were significant. VTC mean values were significantly decreased with the withdrawal of vision, whereas VTC min values decreased with the withdrawal of vision only for the old adults. Further, the VTC mean and min values were significantly reduced for the old compared to the young group.

The VTC mean age × loading interaction revealed that for the old both LWe and RWe induced lower VTC values compared to EqWe. Dyn showed the lowest VTC compared to the remaining loading conditions. For the young group EqWe had significantly higher VTC than RWe. Dyn also showed the lowest VTC compared to all remaining conditions. Finally, post hoc analysis for the VTC min main effect of loading showed that EqWe was significantly higher than Dyn or RWe or LWe.

### Spatial-temporal distributional patterns of VTC

**Figure 4:**
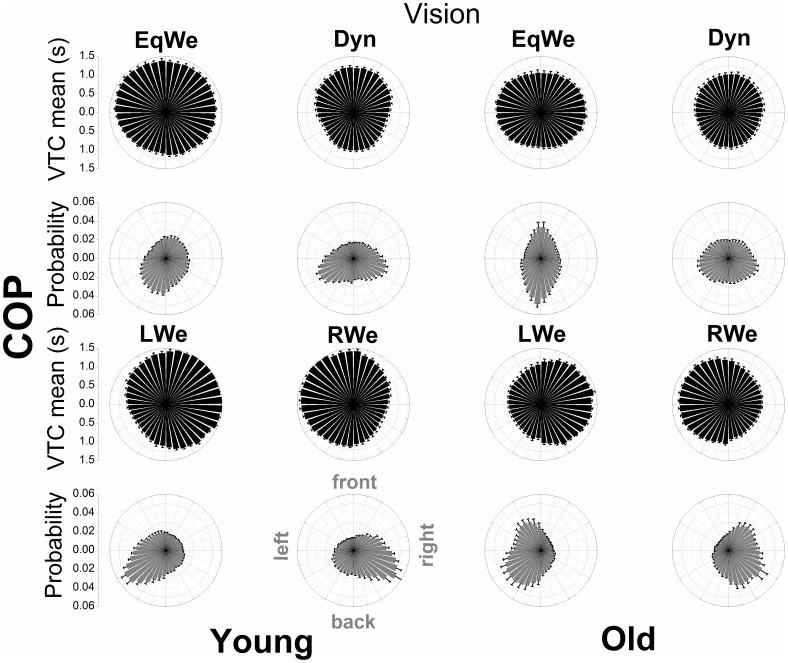
Polar distributions of VTC mean magnitude and probability of a virtual contact across the 40 boundary segments as a function of age and loading (with normal vision) using COP data. Each bar represents the group mean value ± SE (N = 12) for the respective boundary segment.

**Figure 5:**
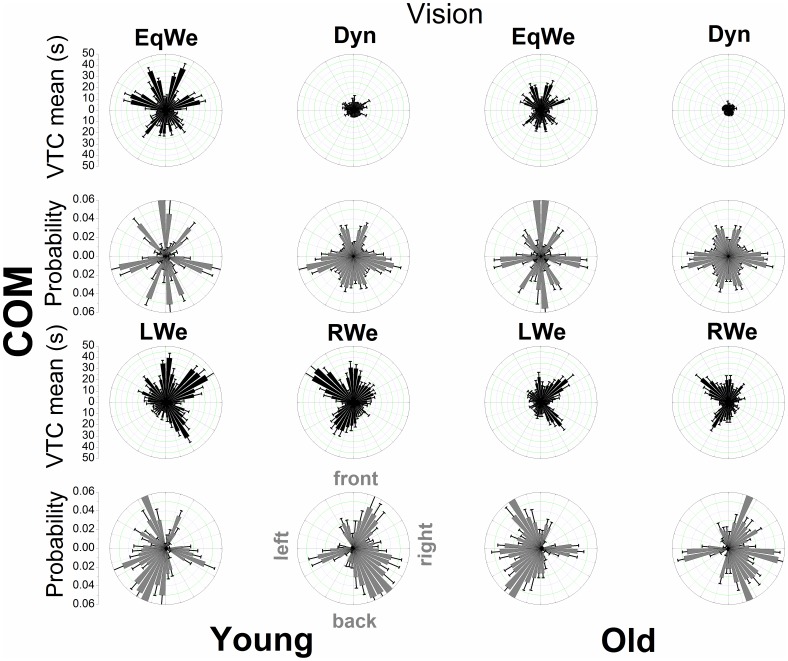
Polar distributions of VTC mean magnitude and probability of a virtual contact across the 40 boundary segments as a function of age and loading (with normal vision) using COM data. Each bar represents the group mean value ± SE (N = 12) for the respective boundary segment.

**Figure 6:**
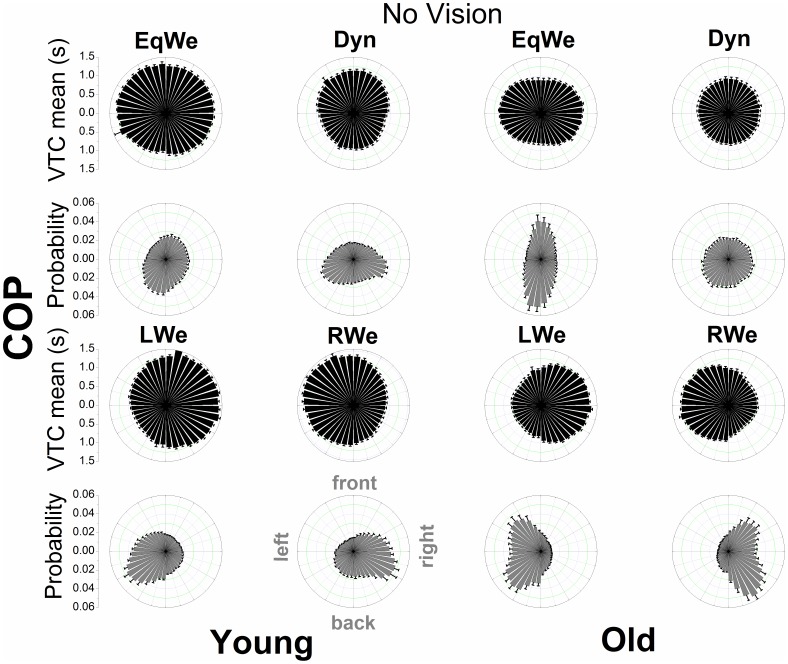
Polar distributions of VTC mean magnitude and probability of a virtual contact across the 40 boundary segments as a function of age and loading (visual information removed) using COP data. Each bar represents the group mean value ± SE (N = 12) for the respective boundary segment.

**Figure 7:**
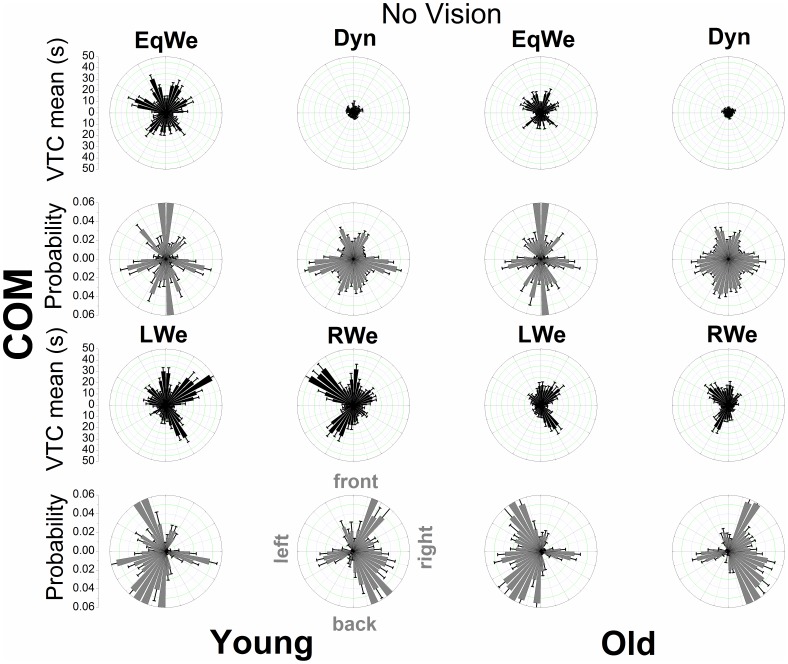
Polar distributions of VTC mean magnitude and probability of a virtual contact across the 40 boundary segments as a function of age and loading (visual information removed) using COM data. Each bar represents the group mean value ± SE (N = 12) for the respective boundary segment.

Figures 4, 5, 6, and 7 show the mean VTC mean values and the mean probability of virtual contacts for each of the 40 boundary segments. The polar distributions are displayed separately for both COP and COM data with vision available and under the removal of vision.

For both COP and COM data there is a clear qualitative change in the shape of the distributions across loading conditions and age groups. When visual information was removed the overall shape of the distributions does not appear to change. In addition, the relatively smooth COP distributions contrast with the COM distributions that show discrete segmented distributions within prevalent spatial regions. The individual distributions of the COM appear to be even sharper.

We examined the general relationship of the mean magnitude of VTC and the probability of a virtual contact for each age group and experimental condition through a quadratic function. For the COP data the significant inverse relationships of VTC and probability were well fitted by a quadratic function (R^2^ values ranging from 0.82 to 0.94), showing that for low VTC mean values the probability of a virtual contact was highest and that this probability was reduced as the mean magnitude of VTC increases. The R^2^ values for the COM ranged from 0.14 to 0.51.

### Complexity (LZC) of Boundary (using COP)

Table 1 shows the results of the Boundary complexity analysis. Furthermore, typical boundary segment sequences are provided in the supplementary video material (Video S1). Age (F_1,22_ = 6.51, p<0.05), vision (F_1,22_ = 12.10, p<0.01) and loading (F_2.08,45.67_ = 19.90, p<0.01) significantly affected LZC. The interactions of age × vision (F_1,22_ = 4.75, p<0.05) and age × loading (F_2.08,45.67_ = 6.02, p<0.01) were also found to be significant. Pairwise comparisons of age × vision showed that only for the old group were the complexity values reduced when vision was removed compared to available vision. The age × loading interaction revealed that the old showed decreased complexity compared to the young adult group during EqWe and LWe. Further, for both age groups both LWe and RWe conditions were less complex than EqWe. In addition, in the young group complexity was significantly lower during Dyn compared to EqWe and LWe.

**Table 1:** Group mean ± SE (N = 12) Lempel Ziv complexity values (LZC) for the Boundary segment sequence for both COP and COM as a function of age, vision and loading.

	Young adults				Old adults			
	EqWe	Dyn	LWe	RWe	EqWe	Dyn	LWe	RWe
Boundary Complexity – Vision								
COP	.78571	.74253	.76697	.75663	.75533	.73772	.73471	.74287
	±.0016	±.0063	±.0043	±.0066	±.011	±.007	±.0086	±.0111
COM	.49753	.60669	.50177	.50170	.50127	.61702	.51608	.51058
	±.0067	±.0048	±.0060	±.0090	±.0060	±.0036	±.0045	±.0056
Boundary Complexity – No Vision								
COP	.78216	.74092	.75886	.75441	.73703	.72816	.71952	.71864
	±.0026	±.0055	±.0045	±.0049	±.0145	±.0076	±.0138	±.0136
COM	.50108	.61981	.51366	.51247	.50635	.61789	.51623	.51557
	±.0084	±.0044	±.0075	±.0065	±.0062	±.0027	±.0054	±.0061

### Complexity (LZC) of Boundary (using COM)

Vision (F_1,22_ = 38.50, p<0.01) and loading (F_3,66_ = 752.00, p<0.01) significantly affected LZC complexity (Tab. 1). The Boundary complexity was higher when removing vision than when vision was available. Post hoc analysis of the main effect of loading showed that all multiple comparisons, except LWe vs. RWe were significant. Complexity was lowest for EqWe and highest for Dyn.

### Surrogate data analysis for Boundary complexity

An additional Boundary complexity analysis was performed with a lower resolution of boundary segments (20 instead of 40 segments) to test for artifacts due to a specific number of boundary segments. The mixed ANOVA showed that the reported findings regarding the Boundary complexity remained unchanged.

In addition, the sequences were randomly shuffled in order to check whether the obtained complexity values resulted from a random process or are due to nonlinearity. The t-tests (COP Boundary: |t_23_| = 27.12; COM Boundary: |t_23_| = 42.15) showed that the shuffled sequences produced throughout significantly higher complexity values, p<0.01.

## Discussion

This study investigated virtual-time-to-contact (VTC) and Lempel-Ziv-Complexity (LZV) in the study of human stance to quantify the risk of potentially losing postural stability (taking a step or falling). We examined the influence of aging, visual information and leg loading on the regulation of upright two-leg stance. In particular, we investigated whether the spatial-temporal distributional patterns for virtually contacting the stability boundary lost complexity [36], [38] with aging and whether these distributions were influenced by the availability of vision and load weighting of the legs. The boundary distributional patterns of VTC provide theoretical insight into the control of posture and hold relevance to the risk of losing postural stability in aging [3], [24].

### Magnitude of postural motion and stability boundary

The COP path length was analyzed as a traditional measure of postural control and indicator of stability. As anticipated the COP path length increased with aging [16], when visual information was removed [37], when posture was more challenged by unequal loading on the legs [32], and during the dynamic in contrast to the quiet standing trials. These results confirm that the overall magnitude of COP displacement reveals how the postural control system handles increasingly difficult constraints to standing still. However, no conclusions in terms of the instantaneous stability can be drawn [17]–[20].

Our results also confirm that the area of the functional stability boundary ellipse decreased in the old compared to young adults [8]. Determining a functional boundary as shown here has the advantage that one can scale the coordinates of the stability boundary to influencing factors such as the availability of vision or aging. It can also help to define the ratio of the region of postural motion and the region of stability, thus emphasizing an interpretation of the amount of postural motion with respect to the individual maximum stability tolerance [17].

### Virtual time-to-contact (VTC) and distributional patterns of VTC with the functional stability boundary

The VTC approach in this study offers one feasible solution to relate postural motion to the individual maximum stability tolerance from a mechanical point of view. This study focused on the temporal safety margin when virtually interpolating the COP or COM to the functional stability boundary. The VTC mean values in particular confirmed previous findings [8], [21], [23], [44], that is, the VTC mean decreased with aging, when visual information was removed and when challenging posture through leg loading - just the reverse trend of the COP path length. The reduced VTC values indicate that the postural control system has a lower temporal safety margin or less potential time for adequately balancing the unstable human body.

A unique approach of the VTC analysis here was the decomposition of VTC into the different spatial regions of the stability boundary in order to quantify and gain deeper insight into the probabilistic properties of losing postural stability. In the old adults during regular two-legged stance (EqWe) the probability distribution of virtual contacts (COP) showed a clear symmetrical bimodal distribution with the peaks at the front and back boundary segments. In particular the probability for the back segments with reduced VTC magnitude was highest for the old adults. The geometry of the feet may drive the channeling of COP motion in AP direction. However, in the young adults these patterns were not replicated, in fact the probability distribution appears to be more uniform. VTC and probability are inversely related; considered together; distributions of the VTC magnitude and the probability of a virtual contact also intuitively link the postural motion to the risk of falls or losing postural stability [1]. The strong inverse relationship between VTC magnitude and probability raises the question as to whether there might be a simple mechanistic relationship between these two quantities. Further research is needed to investigate this relationship.

In the EqWe stance the highest risk of losing postural stability (taking a step or falling) was in the backwards direction. This direction of motion has also been shown to be even more critical or unstable in Parkinson's disease [27] and studies on real-world falls in the elderly have shown that in particular forward and especially backward falls were reported more frequently than sideward falls [3]–[4], [45]. Van Wegen et al. [23] also found the largest effects of aging on VTC during a backward lean condition. The generally increased risk of falling motion in AP direction in the old adults may also indicate an associated postural control strategy that requires less demanding cortical resources [46]. In addition, it should be noted that the lateral postural stability has also been shown to be a valid predictor of future risk of falling in aging adults [47].

In the probability distribution (COP) for loading on either the right (RWe) or left (LWe) leg [31] there was one main peak at the corresponding lateral boundary segments (e.g. left boundary segments for LWe). These patterns possibly emerged, because the postural control system constantly needs to correct for not losing stability to the side the body is inclined to. Interestingly, the corrections towards the other directions are more uniform. In addition, for the elderly the virtual contacts are more broadly distributed along the boundary segments that the body is inclined to which might reflect the need to produce more counterbalanced mechanical stress at the lower extremities or simply an increased imbalance.

We conclude that approaching the complexity of postural dynamics through virtual interpolations to the stability boundary reveals the risk of losing postural stability as a function of the direction of postural motion and that the risk of losing postural stability in any particular direction depends on age [28] and the task constraints to posture. When examining these distributions for the COM many features of the COP distributions seem to be similar, although the distributions for the COM are segmented into discrete regions of the stability boundary. The prevalent regions and dead spaces across the stability boundary segments for COM are attributed to be a reflection of the body's inertia in contrast to the massless COP. It appears that COP is exerting continuous control on discontinuous spatial/temporal dynamics of COM – a postulation that warrants further investigation. The higher COM VTC mean values in contrast to the COP VTC also reflect the damping of the controlled variable, the COM [15].

### Complexity and increased consistency with advanced age

We used the Lempel Ziv compression algorithm [39] to approximate the Kolmogorov complexity of a symbolic sequence representing postural dynamics. We applied this measure to the postural data to quantify not only the information content, but also the consistency in performance. Our proposed analysis of system complexity adds to other sensitive nonlinear tools for postural analysis [19], [48]–[49], however, the computational speed is much faster and it is suitable for on-line implementation for example in medical assistive devices [40], [50].

For the COP the LZC of the Boundary sequence decreased when vision was removed only in the old adults and was also lower in the old compared to the young during EqWe and LWe. It appears that with aging and when visual information is removed the spatial-temporal solutions of the postural control system in exploring the workspace are reduced, supporting the *loss of complexity* with aging hypothesis [36], [38]. Exploring the workspace may keep adaptability high, therefore, the more frequent recurrent patterns in the elderly could be one of the reasons why the ability to adaptively compensate perturbations deteriorates with aging [51]. However, the increased predictability with aging may enhance the possibility to assist postural control with artificial systems [50].

In addition, the complexity generally decreased under the unequal weight loading conditions in contrast to EqWe and was lowest in the Dyn condition. This shows that when task difficulty is higher or when more voluntary directed motion (Dyn) is involved complexity decreases. The Boundary complexity values using the COM were in general lower than those on COP. Also, there was no effect of aging with COM. However, the main effects of vision and loading were similar, except that Boundary complexity was higher when vision was removed and lowest for EqWe and highest for Dyn. This finding is the reverse of the VTC COP complexity, which has been attributed to be a reflection of damping mechanisms in human upright stance [15].

Finally, it should be noted that our previous work in isometric force control has shown a bidirectional change of movement complexity in aging adults [36] as a function of task and the attractor dynamics of force output, opening the conjecture that the general issue may be loss of adaptability in aging rather than necessarily a loss of complexity. The findings here in the quiet stance EqWe condition showed the aging loss of complexity effect. There was not, however, an increment of complexity in VTC at the boundary in the dynamic postural task (Dyn) as would be expected from the parallels to the intrinsic dynamics of the isometric force conditions [36]. This may be because the dynamics of the time series of the amplitude of COP or COM motion was not analyzed as was in effect the case in the isometric force studies.

### Concluding remarks on VTC boundary control framework for falls assessment

This study revealed that VTC as a time function based on COP/COM dynamics provides a basis to assess future instability in terms of temporal-spatial risk of losing postural stability and the direction of potentially losing postural stability. The computational cost of the implemented algorithms is low and thus suitable for on-line applications [40]. We found that aging, vision and the postural task are critical in determining the characteristics of the risk of losing postural stability. In particular, we showed that the instantaneous postural stability is compromised in aging adults and that the complexity of the postural dynamics, as estimated by LZC of VTC is decreased in aging. Broader impacts of our research include the possibility of implementing the here introduced method of analyzing VTC in the clinical setting [3], [45] for assessing instantaneous postural instability in relation to the functional stability boundary.

## Supporting Information

Video S1 **A video animation supports this paper and provides more details on VTC computation and subsequent data analysis.**
(MP4)
